# Bisphosphonates Preserve Bone Mineral Density and Suppress Bone Turnover Markers in Early Menopausal Women: A Systematic Review and Meta‐Analysis of Randomized Trials

**DOI:** 10.1002/jbm4.10748

**Published:** 2023-04-14

**Authors:** Aria Ahadzadeh Ardebili, Timothy Fu, Nicole Dunnewold, Fariba Aghajafari, Emma O. Billington

**Affiliations:** ^1^ Cumming School of Medicine University of Calgary Calgary Alberta Canada; ^2^ Health Sciences Library University of Calgary Calgary Alberta Canada; ^3^ McCaig Institute for Bone & Joint Health University of Calgary Calgary Alberta Canada

**Keywords:** ANTIRESORPTIVES, CLINICAL TRIALS, DXA, MENOPAUSE, OSTEOPOROSIS

## Abstract

Most women do not qualify for pharmacologic osteoporosis treatment until more than a decade after menopause, by which time they will have lost up to 30% of their bone mass and may have already sustained fractures. Short or intermittent courses of bisphosphonate therapy, initiated around the time of menopause, might prevent excessive bone loss and lower long‐term fracture risk. We undertook a systematic review and meta‐analysis of randomized controlled trials (RCTs) to determine the effects of nitrogen‐containing bisphosphonates on fracture incidence, bone mineral density (BMD), and bone turnover markers in early menopausal women (ie, perimenopausal or <5 years postmenopausal) over ≥12 months. Medline, Embase, CENTRAL, and CINAHL were searched in July 2022. Risk of bias was evaluated using the Cochrane Risk of Bias 2 tool. Random effect meta‐analysis was undertaken using RevMan v5.3. In total, 12 trials were included (*n* = 1722 women); five evaluated alendronate, three risedronate, three ibandronate, and one zoledronate. Four were at low risk of bias; eight raised some concerns. Fractures were infrequent in the three studies that reported them. Compared with placebo, bisphosphonates improved BMD over 12 months (mean percentage difference, 95% confidence interval [CI]) at the spine (4.32%, 95% CI, 3.10%–5.54%, *p* < 0.0001, *n* = 8 studies), the femoral neck (2.56%, 95% CI, 1.85%–3.27%, *p* = 0.001, *n* = 6 studies), and the total hip (1.22%, 95% CI 0.16%–2.28%, *p* = 0.002, *n* = 4 studies). Over treatment durations of 24 to 72 months, bisphosphonates improved BMD at the spine (5.81%, 95% CI 4.71%–6.91%, *p* < 0.0001, *n* = 8 studies), femoral neck (3.89%, 95% CI 2.73%–5.05%, *p* = 0.0001, *n* = 5 studies) and total hip (4.09%, 95% CI 2.81%–5.37%, *p* < 0.0001, *n* = 4 studies). Bisphosphonates reduced urinary N‐telopeptide (−52.2%, 95% CI −60.3% to −44.2%, *p* < 0.00001, *n* = 3 studies) and bone‐specific alkaline phosphatase (−34.2%, 95% CI −42.6% to −25.8%, *p* < 0.00001, *n* = 4 studies) more than placebo at 12 months. This systematic review and meta‐analysis shows that bisphosphonates improve BMD and lower bone turnover markers in early menopause, warranting further investigation of these agents for osteoporosis prevention. © 2023 The Authors. *JBMR Plus* published by Wiley Periodicals LLC on behalf of American Society for Bone and Mineral Research.

## Graphical abstract

This systematic review and meta‐analysis of randomized controlled trials demonstrates that nitrogen‐containing bisphosphonates improve bone density and lower bone turnover markers in early menopausal women (i.e. perimenopausal or <5 year postmenopausal), warranting further investigation of these agents for osteoporosis prevention.

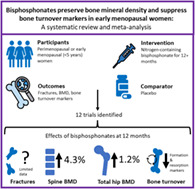

## Introduction

Bisphosphonate medications can preserve bone mineral density (BMD) and reduce fracture risk in older women at high risk of fracture and are considered first‐line pharmacologic treatments for postmenopausal osteoporosis.^(^ ^1^, ^2^ ^)^ However, although women lose up to 30% of their bone mass around the time of menopause and in the decade that follows,^(^ ^3^, ^4^, ^5^ ^)^ most do not meet recommended thresholds for bisphosphonate therapy until they are at least 65 years old,^(^ ^1^, ^6^, ^7^, ^8^, ^9^, ^10^, ^11^ ^)^ by which time extensive irreversible bone loss and/or fractures have already occurred.^(^ ^12^, ^13^, ^14^, ^15^ ^)^ This management paradigm is reactive and not always aligned with patient preferences: many women wish to take a proactive approach, initiating pharmacologic therapy before experiencing a fracture or reaching a high‐risk status.^(^ ^16^ ^)^

Given the long skeletal half‐life of nitrogen‐containing bisphosphonates,^(^ ^17^, ^18^, ^19^ ^)^ there has been recent interest in preventative treatment with either intermittent short courses of oral bisphosphonates or infrequently dosed intravenous zoledronate,^(^ ^20^ ^)^ beginning at the time of menopause. Modeling analyses indicate that a preventative strategy in which zoledronate is administered every 5 years, starting at age 50 years, could substantially reduce the future burden of osteoporosis and fragility fracture, but empirical data are lacking.^(^ ^21^ ^)^ The pivotal clinical trials of nitrogen‐containing bisphosphonates were conducted in older populations (age > 60 years), most with established osteoporosis or prior fractures,^(^ ^1^ ^)^ and although bisphosphonates are also approved for osteoporosis prevention, the effects of these agents in perimenopausal and early postmenopausal women are not as well studied. It is possible that the skeletal response to bisphosphonate therapy would be even more profound in perimenopause and early postmenopause, as the perimenopausal and early postmenopausal years are a time of excessive bone resorption^(^ ^22^ ^)^ and bisphosphonates are preferentially incorporated into the skeleton at sites of increased bone turnover.^(^ ^23^ ^)^ Some trials have examined the effects of nitrogen‐containing bisphosphonates on bone health in women who are either perimenopausal^(^ ^24^, ^25^ ^)^ or <5 years postmenopausal,^(^ ^26^, ^27^, ^28^, ^29^, ^30^, ^31^, ^32^, ^33^, ^34^ ^)^ demonstrating that bisphosphonates can increase BMD and reduce bone turnover markers in this population. However, these studies do not provide clarity regarding whether the effects of bisphosphonates are dependent on bisphosphonate type, duration of therapy, or baseline characteristics of participants. Therefore, our objective was to systematically review existing randomized, placebo‐controlled trials of nitrogen‐containing bisphosphonate agents, conducted in early menopausal women and to determine the effects of these agents on fracture incidence, BMD, and bone turnover markers.

## Methods

### Registration

This review was prospectively registered on the International Prospective Register of Systematic Reviews (PROSPERO CRD42020169109).

### Eligibility criteria

Inclusion criteria were as follows: (i) randomized controlled trials conducted in ambulatory, community‐dwelling women in early menopause (ie, either perimenopause or within 5 years of their final menstrual period or surgical menopause); trials that also included older women were eligible as long as they conducted prespecified subgroup analyses of early menopausal women; (ii) study intervention consisted of treatment with a nitrogen‐containing bisphosphonate (risedronate, alendronate, ibandronate, or zoledronate); (iii) study comparator consisted of a placebo; (iv) intervention period was at least 12 months; (v) reported outcomes included one or more of: fracture incidence, BMD at the lumbar spine, femoral neck, or total hip (assessed using dual‐energy x‐ray absorptiometry [DXA]), validated bone turnover markers (C‐terminal telopeptide [CTX], N‐terminal telopeptide [NTX], type 1 procollagen N‐terminal propeptide [P1NP], bone‐specific alkaline phosphatase [bsALP]).

Studies were excluded if they assessed animals, men, premenopausal women, women >5 years since their final menstrual period (with no prespecified analysis of an early menopausal subgroup), trial cohorts with conditions known to cause secondary osteoporosis and/or likely to impact on bone turnover (hyperparathyroidism, hyperthyroidism, malabsorptive diseases, renal disease, hepatic disease, glucocorticoid therapy), or individuals who had been previously treated with osteoporosis pharmacotherapy (bisphosphonates, estrogen, raloxifene, denosumab, teriparatide). Concurrent supplementation with calcium and/or vitamin D, and concurrent exercise programs were not criteria for exclusion, provided that the concurrent therapy was the same for bisphosphonate and placebo arms.

### Information sources

Our team, which included a senior librarian (ND), developed a search strategy to identify randomized controlled trials which compared the effects of a nitrogen‐containing bisphosphonate intervention with placebo in early menopausal women (ie, in perimenopause or within 5 years of final menstrual period at the time of study enrollment) on BMD or bone turnover markers over a follow‐up duration of 12 or more months. Excerpta Medical Database (EMBASE), MEDLINE, Cochrane Central Register of Controlled Trials (CENTRAL), and CINAHL were searched for English‐language studies published from the time of inception up until the search date. In addition, clinical trials registries (www.clinicaltrials.gov, www.controlled-trials.com/mrct, www.anzctr.org.au, Health Canada Clinical Trials Database, WHO clinical trial registry [ICTRP]) and the past 3 years of conference abstracts (identified using the “conference” filter on Web of Science) were searched to identify ongoing or recently completed trials without corresponding full‐text publications. Reference lists of all identified primary articles and relevant reviews were hand searched by an osteoporosis content expert (EOB) for additional eligible studies. A full search strategy is provided in the online Supporting Information.

### Selection process

Titles and abstracts of all articles identified by the literature search were screened independently by two investigators (AAA and TF), and those that appeared to meet eligibility criteria were flagged for full‐text review. Any discrepancies in title and abstract review were resolved via discussion with a third investigator (EOB). In the case of articles which appeared to meet inclusion criteria but for which information to confirm eligibility was not available (eg, no full‐text publication), study authors were contacted to request the necessary information regarding eligibility. If this information could not provided by the author, the study was excluded.

Articles selected for full‐text review were evaluated independently by two investigators (AAA and TF) in order to determine eligibility. Discrepancies were again resolved via discussion with two additional investigators (EOB and FA). Reviewer agreement for title and abstract screening and for full‐text evaluation was calculated using Cohen's kappa (κ) statistic. Study selection was done using Covidence systematic review software (Veritas Health Innovation, Melbourne, Australia).

### Data collection process

A standardized and pilot‐tested extraction form was used by two independent reviewers (AAA and TF) to obtain relevant data and checked by a third investigator (EOB). The following data were extracted for each included study: general information (title, authors, publication source, publication year), trial characteristics (design, setting, inclusion and exclusion criteria, randomization method, allocation procedure, blinding of participants, reporting of outcome data, withdrawals and dropouts, intention‐to‐treat analysis), participant characteristics (age, sex, relevant comorbidities and medications, baseline BMD, baseline bone turnover markers), intervention characteristics (type of nitrogen‐containing bisphosphonate, dosage, mode of administration, frequency, and duration of treatment), relevant cointerventions (calcium and vitamin D supplementation, exercise), and outcomes assessed during intervention period. We extracted data for all relevant outcomes assessed at 12 months, and where available, the longest assessment interval of >12 months. Where data were presented in figure form only, an image processing program (ImageJ; National Institutes of Health, Bethesda, MD, USA) was used to determine means and standard deviations or standard errors. Missing data were requested from study authors via email.

### Study risk of bias

Two investigators (EOB and FA) independently assessed each study for risk of bias using the Risk of Bias Tool 2.0 published by the Cochrane Collaboration,^(^ ^35^ ^)^ which evaluates the following five domains: bias arising from the randomization process, bias due to deviations from intended interventions, bias due to missing outcome data, bias in measurement of the outcome, and bias in selection of the reported result. An overall risk of bias judgment was made for each study in accordance with recommendations from the Cochrane Collaboration.^(^ ^35^ ^)^ Disputes regarding overall risk of bias were resolved via consensus.

### Effect measures

The primary outcome was initially specified as the number of individuals who experience one or more low‐trauma fractures during the follow‐up period. However, none of the identified studies were designed to assess fracture incidence and only three eligible studies reported on fractures; therefore, we were unable to include this outcome in our quantitative synthesis. Prespecified secondary outcomes were: percentage change in BMD at the lumbar spine, femur neck and total hip, distal radius, and whole body between baseline and 12 months, and percentage changes in bone turnover markers (CTX, NTX, P1NP, bsALP) between baseline and 12 months. Prespecified analyses also included an evaluation of the effects of bisphosphonate therapy on BMD and bone turnover markers in studies lasting >12 months. Exploratory subgroup analyses were planned to assess the effect of baseline BMD (ie, osteoporosis or not) and type of bisphosphonate on BMD and bone turnover outcomes.

### Synthesis methods

For trials which compared multiple bisphosphonate doses, we assessed only the doses corresponding to approved dosing regimens for the treatment and prevention of osteoporosis in the United States, as summarized in recently published guidelines (ie, risedronate 5 mg/day, 35 mg/week, or 150 mg/month, alendronate 5–10 mg/day or 35–70 mg/week, oral ibandronate 2.5 mg/day, 150 mg/month, or 3 mg intravenously [IV] every 3 months [q3 months], IV zoledronate 5 mg every 1 to 2 years [q1–2 years]).^(^ ^10^ ^)^ Where relevant, data were pooled as recommended in the Cochrane Handbook.^(^ ^36^ ^)^ Missing standard deviations were calculated from standard errors or confidence intervals as demonstrated in the Cochrane Handbook.^(^ ^36^ ^)^ When an article did not provide adequate information to include a relevant outcome in a quantitative synthesis, we contacted the study authors for additional information. If it was not possible to obtain standard deviations through calculation or from the study authors, they were imputed.^(^ ^36^ ^)^ Data from included studies were summarized narratively, and where appropriate, meta‐analysis was undertaken using an inverse variance random‐effects model. To evaluate for heterogeneity among included studies, the *I* ^2^ heterogeneity statistic was calculated. An *I* ^2^ of 0% to 40% is suggestive of low heterogeneity, 30% to 60% indicates moderate heterogeneity, and > 60% indicates substantial heterogeneity. RevMan (Version 5.3; The Cochrane Collaboration, 2020) was used for data analysis.

### Reporting bias assessment

To determine the impact of publication and reporting bias on our study selection, we planned to create funnel plots for any outcome containing 10 or more studies by plotting the effect for each trial by the inverse of its standard error and assessing for visual symmetry. We also planned to assess the impact of publication bias mathematically for outcomes with 10 or more studies using Begg's test. For outcomes containing fewer than 10 studies, formal assessment of reporting bias was not undertaken, in accordance with recommendations from the Cochrane Handbook.^(^ ^36^ ^)^

### Certainty assessment

An overall summary of the strength of the evidence that nitrogen‐containing bisphosphonates maintain BMD, reduce bone turnover, and lower fracture incidence in perimenopausal and early menopausal women was made using the Grading of Recommendations, Assessment, Development and Evaluations (GRADE) framework.^(^ ^37^ ^)^

## Results

### Study selection

**Fig. 1:**
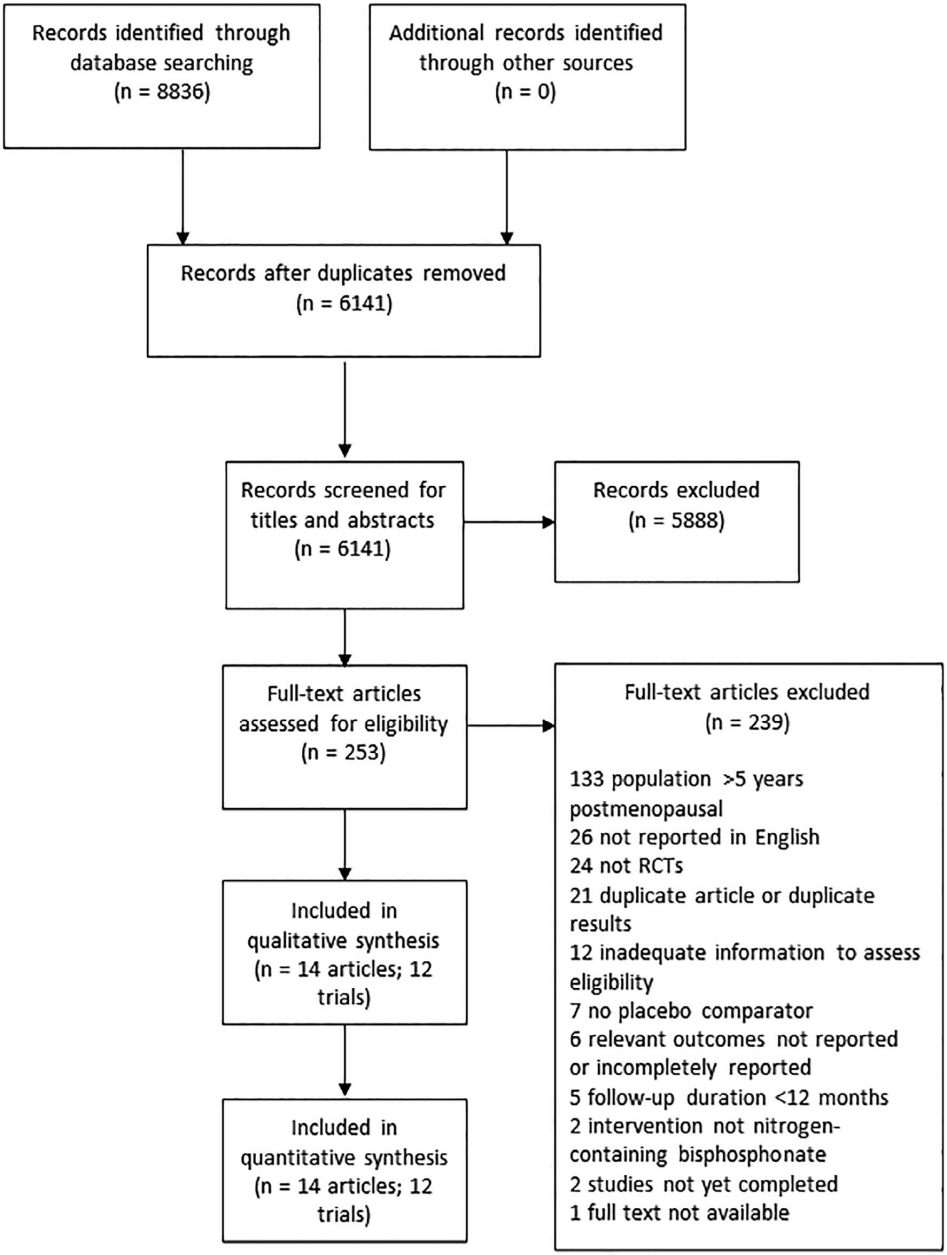
Article identification process.

A literature search was conducted on April 20, 2020 and updated on July 29, 2022. As shown in Fig. 1, our search identified 8836 records, of which 2695 were duplicates. Titles and abstracts of 6141 records were screened. Of the 253 records that met criteria for full‐text review, 223 were excluded on the basis of full‐text review for reasons shown in Fig. 1. Fourteen articles reporting on 12 unique trials were selected for inclusion.^(^ ^24^, ^25^, ^26^, ^27^, ^28^, ^29^, ^30^, ^31^, ^32^, ^33^, ^34^, ^38^, ^39^ ^)^ Twelve were full‐text journal articles, one was a clinical trial registration page^(^ ^39^ ^)^ that presented results from an early postmenopausal subgroup analysis not reported in the corresponding full‐text article,^(^ ^32^ ^)^ and one was an industry website which presented results from the trial not published elsewhere.^(^ ^40^ ^)^ One of the included articles enrolled women who were up to 6 years postmenopausal, although the corresponding author was contacted and confirmed that all participants were within 5 years of menopause.^(^ ^34^ ^)^

Agreement between reviewers was 95.7% for the title and abstract screen (κ = 0.60) and 98.8% for the full‐text screen (κ = 0.89).

### Study characteristics

Characteristics of the 12 included trials (*n* = 1722 women) are set out in Table 1. Each of these studies randomized eligible women to receive placebo or a nitrogen‐containing bisphosphonate at an eligible dose and had data available for at least one relevant outcome. All included trials were industry‐funded and conducted in a community setting. Two trials enrolled only perimenopausal women,^(^ ^24^, ^25^ ^)^ five enrolled only women who were within 5 years of their final menstrual period,^(^ ^26^, ^27^, ^33^, ^34^ ^)^ and the remaining five trials included a subgroup analysis of women who were within 5 years of their final menstrual period.^(^ ^28^, ^29^, ^30^, ^31^, ^32^, ^38^, ^39^ ^)^ No studies included women with established osteoporosis, although low bone density (ie, “osteopenia”) was an inclusion criterion for two studies^(^ ^24^, ^32^ ^)^ and three studies performed subgroup analyses of women with low bone density.^(^ ^28^, ^29^, ^31^ ^)^ Five trials reported on the ethnicity of the participants; as shown in Table 1, the vast majority of participants in these trials were white.^(^ ^24^, ^25^, ^27^, ^32^, ^40^ ^)^ Alendronate was evaluated in five trials,^(^ ^24^, ^25^, ^26^, ^30^, ^34^, ^38^ ^)^ risedronate in three,^(^ ^27^, ^33^, ^40^ ^)^ ibandronate in three,^(^ ^28^, ^29^, ^31^ ^)^ and zoledronate in one.^(^ ^32^, ^39^ ^)^ Follow‐up ranged from 12 to 72 months.

**Table 1:** Summary of Randomized Controlled Trials Assessing the Effects of Nitrogen‐Containing Bisphosphonates on Skeletal Parameters in Early Menopausal Women

Trial	Design	Duration	Population	Location	n/N^a^	Mean age	Outcomes assessed	Funding
McClung and colleagues^(^ ^26^ ^)^ (1998) Lehmann and colleagues^(^ ^38^ ^)^ (2002) (substudy)	5 arm (1:1:1:1:1) ALN 1 mg/d PO **ALN 5 mg/d PO** ^b^ **ALN 10 mg/d PO** ALN 20 mg/d* PO PBO	36	Early postmenopausal No OP Community‐dwelling Ethnicity NR	15 centers worldwide	447/447 ALN 5 or 10 mg/d and PBO: 266 63 (subgroup)	51–52 53	LS BMD FN BMD TH BMD NTX bsALP CTX	Industry
Mortensen and colleagues^(^ ^27^ ^)^ (1998)	3 arm (1:1:1) **RIS 5 mg/d PO** RIS cyclic PO PBO	24 m 12 m off	Early postmenopausal No OP Community‐dwelling 100% White ethnicity	2 centers (USA, Denmark)	111/111 Ris 5 mg/d and PBO: 73	51–52	Fracture LS BMD FN BMD	Industry
Stakkestad and colleagues^(^ ^28^ ^)^ (2003)	4 arm (1:1:1:1) IBN 0.5 mg/3 m IV IBN 1 mg/3 m IV **IBN 2 mg/3 m IV** PBO	12 m	Postmenopausal No OP Community‐dwelling Early postmenopausal subgroup (1‐3y) Ethnicity NR	Multiple centers in Norway, Russia, Czech Republic	294/629 IBN 2 mg/3 m and PBO: 146	NR	LS BMD FN BMD TH BMD CTX bsALP	Industry
Tanko and colleagues^(^ ^29^ ^)^ (2003)	4 arm (1:1:1:1) IBN 5 mg/wk PO IBN 10 mg/wk PO **IBN 20 mg/wk PO**	24 m	Postmenopausal No OP Community‐dwelling Early postmenopausal subgroup (1‐3y) Ethnicity NR	11 European centers	290/630 IBN 20 mg/wk and PBO: 147	NR	LS BMD TH BMD CTX bsALP	Industry
McClung and colleagues^(^ ^30^ ^)^ (2004a)	3 arm (1:1:1.5) ALN 2.5 mg/d PO **ALN 5 mg/d PO** PBO	72 m	Postmenopausal No OP Community‐dwelling Early postmenopausal subgroup (1‐3y) Ethnicity NR	4 centers in USA, UK, Denmark	NR/1609 ALN 5 mg/d and PBO: 129	NR	LS BMD	Industry
McClung and colleagues^(^ ^31^ ^)^ (2004b)	4 arm (1:1:1:1) IBN 0.5 mg/d PO IBN 1 mg/d PO **IBN 2.5 mg/d PO** PBO	24 m	Postmenopausal No OP Community‐dwelling Early postmenopausal subgroup (1‐3y) Ethnicity NR	11 centers in USA and Canada	311/653 IBN 2.5 mg/d and PBO: 153	NR	LS BMD	Industry
Hooper and colleagues^(^ ^33^ ^)^ (2005)	3 arm (1:1:1) RIS 2.5 mg/d PO **RIS 5 mg/d PO** PBO	24 m	Early postmenopausal No OP Community‐dwelling Ethnicity NR	11 centers in Australia	383/383 Ris 5 mg/d and PBO: 255	53	Fractures LS BMD FN BMD bsALP	Industry
NCT00402441^(^ ^40^ ^)^ (2006)	2 arm (1:1) RIS 35 mg/wk PO PBO	12 m	Early postmenopausal (<5y) No OP 81% White ethnicity	18 centers in US	280/280	NR	LS BMD FN BMD TH BMD	Industry
McClung and colleagues^(^ ^32^ ^)^ (2009) NCT00132808^(^ ^39^ ^)^ (2005)	3 arm (1:1:1) ZOL 5 mg/y IV x1 ZOL 5 mg/y IV x2 PBO	24 m	Postmenopausal Low BMD, no OP Community‐dwelling Early postmenopausal subgroup (<5y) 90% White ethnicity	15 centers	224/581	54	LS BMD FN BMD TH BMD CTX P1NP bsALP	Industry
Burghardt and colleagues^(^ ^34^ ^)^ (2010)	ALN 70 mg/wk PO PBO	24 m	Postmenopausal (1‐6y) Community‐dwelling Ethnicity NR	Single center in USA	53/53	55–56	LS BMD TH BMD NTX bsALP	Industry and non‐industry
Mersereau and colleagues^(^ ^25^ ^)^ (2010)	2 arm (2:1) ALN 70 mg/wk PO PBO	12 m	Perimenopausal No BMD criteria Community‐dwelling 61% White ethnicity	Single center in USA	45/45	49	LS BMD TH BMD NTX bsALP	Industry
Khan and colleagues^(^ ^24^ ^)^ (2014)	2 arm (1:1) ALN 70 mg/wk PO PBO	12 m	Perimenopausal Low BMD Community‐dwelling 90% White ethnicity	Canada; number of centers not reported	45/45	49–50	Fractures LS BMD FN BMD TH BMD NTX bsALP	Industry and non‐industry

^a^ n/N = number of eligible (ie, perimenopausal or <5 years menopausal) women randomized/total number of women randomized; in cases where the study included >1 eligible treatment/dosing arm, the total number of eligible women randomized to the eligible treatment arm(s) and the placebo arm is also indicated.

^b^ Only doses currently approved for use in osteoporosis management were included in analyses; these doses are bolded.

Abbreviation: ALN = alendronate; BMD = bone mineral density; bsALP = bone specific alkaline phosphatase; CTX = C‐terminal telopeptide; Ex = exercise; FN = femoral neck; IBN = ibandronate; IV = intravenous; LS = lumbar spine; m = months; NR = not reported; NTX = N‐terminal telopeptide; OP = osteoporosis; P1NP = procollagen type 1 N‐terminal propeptide; PBO = placebo; PO = oral; rad = radius; RIS = risedronate; TH = total hip; WB = whole body (same as total body); ZOL = zoledronate;

Three trials reported fracture incidence.^(^ ^24^, ^27^, ^33^ ^)^ All trials reported on BMD, of which seven had missing results.^(^ ^26^, ^29^, ^30^, ^31^, ^32^, ^34^, ^39^, ^40^ ^)^ When the authors of these studies were contacted to request the required data, two did not respond,^(^ ^29^, ^40^ ^)^ four indicated that the data were not available,^(^ ^26^, ^30^, ^31^, ^32^, ^39^ ^)^ and one provided the data.^(^ ^34^ ^)^ Regarding bone turnover markers, seven trials either had missing results^(^ ^25^, ^28^, ^30^, ^32^, ^34^, ^38^, ^39^ ^)^ or did not provide enough information to conduct a quantitative analysis.^(^ ^26^, ^29^ ^)^ When the authors of these studies were contacted, three did not respond,^(^ ^28^, ^29^, ^38^ ^)^ three indicated that the data were not available,^(^ ^25^, ^26^, ^30^, ^32^, ^39^ ^)^ and one provided the requested data.^(^ ^34^ ^)^ Ultimately, we obtained 12‐month BMD changes at the lumbar spine from eight trials,^(^ ^24^, ^25^, ^26^, ^27^, ^28^, ^33^, ^34^, ^40^ ^)^ femoral neck from six trials,^(^ ^24^, ^26^, ^27^, ^28^, ^33^, ^34^ ^)^ and total hip from four trials.^(^ ^24^, ^25^, ^28^, ^34^ ^)^ Only one trial reported 12 month changes in total body BMD,^(^ ^26^ ^)^ and only one reported 12 month changes in radius BMD.^(^ ^34^ ^)^ Bone turnover changes at 12 months were available from three trials for NTX^(^ ^24^, ^26^, ^34^ ^)^ and four trials for bsALP.^(^ ^24^, ^26^, ^33^, ^34^ ^)^ No trials reported changes in CTX or P1NP at 12 months.

### Risk of bias in studies

As shown in Table 2, four trials were judged to be at overall low risk of bias^(^ ^28^, ^29^, ^32^, ^33^, ^39^ ^)^ and eight raised some concerns of bias.^(^ ^24^, ^25^, ^26^, ^27^, ^30^, ^31^, ^34^, ^38^, ^40^ ^)^ The most common source of potential bias was missing outcome data (either >10% missing data points or number of missing data points not reported). No studies were deemed to be at high risk of bias, although to our knowledge, the results of one study, which were only available from an industry website, had not undergone any form of peer review.^(^ ^40^ ^)^

**Table 2:** Risk of Bias Assessment of Randomized Controlled Trials Assessing the Effects of Nitrogen‐Containing Bisphosphonates on Skeletal Parameters in Early Menopausal Women

Trial	Domain 1: Bias arising from randomization	Domain 2: Bias due to deviations from intended interventions	Domain 3: Bias due to missing outcome data	Domain 4: Bias in measurement of the outcome	Domain 5: Bias in selection of the reported result	Overall risk of bias judgment
McClung and colleagues^(^ ^26^ ^)^ (1998) Lehmann and colleagues^(^ ^38^ ^)^ (2002) (substudy)	Low	Some concerns (unclear whether they performed an ITT analysis)	Some concerns (>10% of 3y data missing for primary outcome in some groups)	Low	Low (although unclear whether analysis plan was prespecified)	Some concerns
Mortensen and colleagues^(^ ^27^ ^)^ (1998)	Low	Low	Some concerns (>10% of participants did not complete first year of study)	Low	Low	Some concerns
Stakkestad and colleagues^(^ ^28^ ^)^ (2003)	Low	Low	Low	Low	Low	Low risk of bias
Tanko and colleagues^(^ ^29^ ^)^ (2003)	Low	Low	Low	Low	Low	Low risk of bias
McClung and colleagues^(^ ^30^ ^)^ (2004a)	Low	Low	Some concerns (unclear how many participants are missing from analysis)	Low	Some concerns (only LS BMD reported for early postmenopausal subgroup)	Some concerns
McClung and colleagues^(^ ^31^ ^)^ (2004b)	Low (but randomization process not described)	Low	Low	Low	Some concerns (only LS BMD reported for early postmenopausal subgroup)	Some concerns
Hooper and colleagues^(^ ^33^ ^)^ (2005)	Low	Low	Low	Low	Low	Low risk of bias
NCT00402441^(^ ^40^ ^)^ (2006)	Low	Low	Low	Low	Low	Some concerns
McClung and colleagues^(^ ^32^ ^)^ (2009) NCT00132808^(^ ^39^ ^)^ (2005)	Low	Low (although 14.9% of ZOL ×1 withdrew compared to 9% of ZOL ×2 and 7% of placebo group)	Low	Low	Low	Low risk of bias
Burghardt and colleagues^(^ ^34^ ^)^ (2010)	Low (but randomization process not described)	Some concerns (>50% dropout in placebo group and 32% in alendronate group)	Some concerns (high attrition)	Low	Low	Some concerns
Mersereau and colleagues^(^ ^25^ ^)^ (2010)	Some concerns (no info about randomization process)	Low	Low (but 9 participants withdrew before 6 month DXA and were not analyzed)	Low	Low	Some concerns
Khan and colleagues^(^ ^24^ ^)^ (2014)	Low	Some concerns (used paired mean differences, suggesting per protocol analysis)	Low	Low	Low	Some concerns
						

Abbreviation: BMD = bone mineral density; DXA = dual‐energy x‐ray absorptiometry; ITT = intention to treat; LS = lumbar spine; ZOL = zoledronate.

### Fracture outcomes

Fracture outcomes were reported by three trials^(^ ^24^, ^27^, ^33^ ^)^; these data were not appropriate for meta‐analysis. In the study by Mortensen and colleagues,^(^ ^27^ ^)^ one participant in the risedronate 5 mg/day group experienced a vertebral fracture 12 months after discontinuing risedronate and no participants experienced nonvertebral fractures. In the placebo group, there were no vertebral fractures, but three participants experienced fractures of the fingers or hands. Hooper and colleagues^(^ ^33^ ^)^ reported incident vertebral fractures in 7.7% of women receiving risedronate 5 mg/day and 8.3% of women in the placebo group throughout 24 months of treatment. Incidence of nonvertebral fracture was 3.9% in the risedronate 5 mg/day group and 4.8% in the placebo group. Khan and colleagues^(^ ^24^ ^)^ reported no fractures in either the alendronate or placebo group in their trial.

### BMD outcomes

**Fig. 2:**
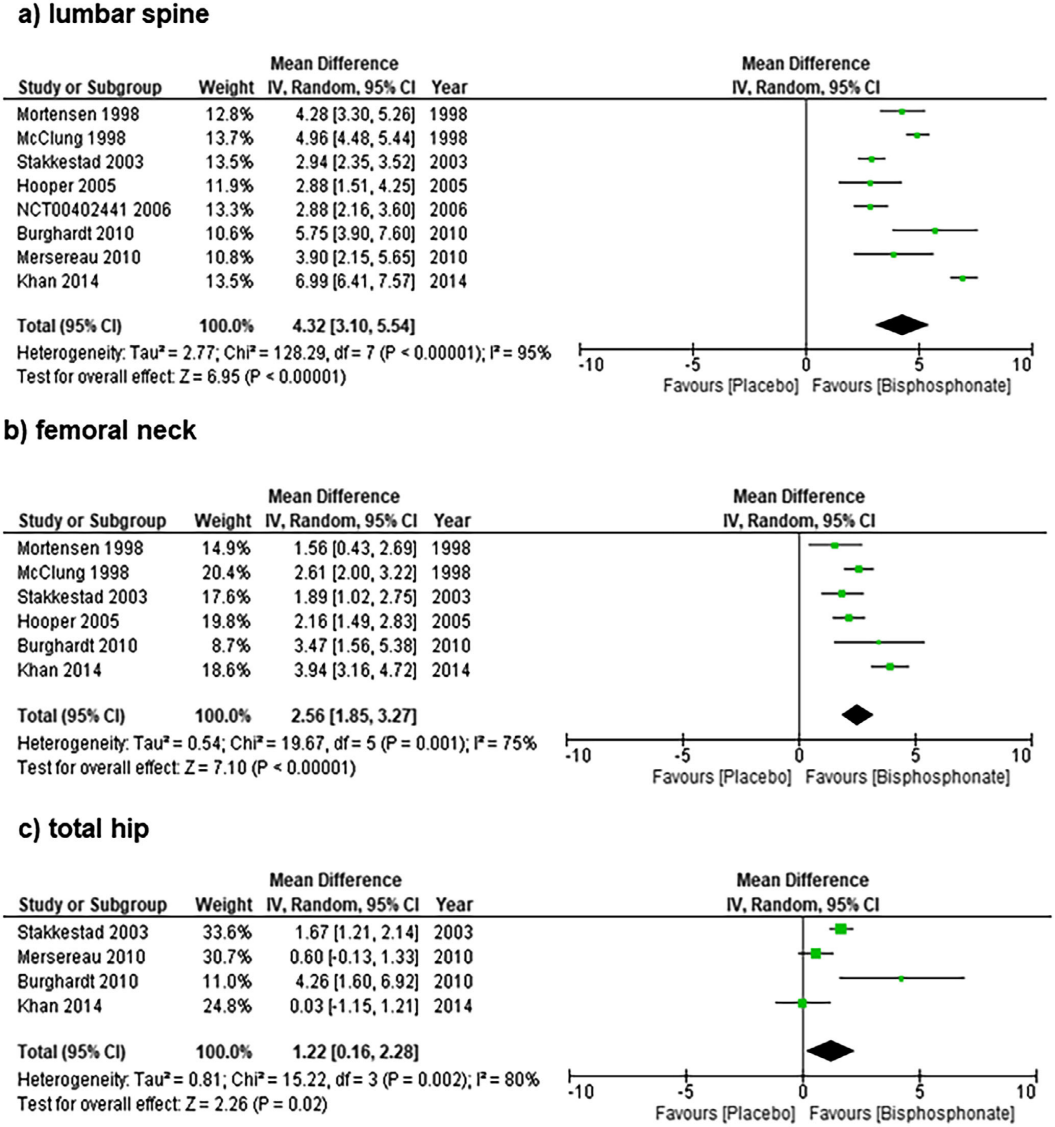
Pooled results demonstrate mean (95% confidence interval [CI]) percent change in BMD of 4.32% (95% CI, 3.10%–5.54%) at the lumbar spine (Fig. 2a), 2.56% (95% CI, 1.85%–3.27%) at the femoral neck (Fig. 2b), and 1.22% (95% CI, 0.16%–2.28%) at the total hip (Fig. 2c).

BMD changes over 12 months of follow‐up are shown in Fig. 2. Pooled results demonstrate mean (95% confidence interval [CI]) percent change in BMD of 4.32% (95% CI, 3.10%–5.54%) at the lumbar spine, 2.56% (95% CI, 1.85%–3.27%) at the femoral neck, and 1.22% (95% CI, 0.16%–2.28%) at the total hip. Heterogeneity was high for all pooled analyses at 12 months (*I* ^2^ 75%–95%). The study^(^ ^26^ ^)^ that reported on change in BMD at the total body at 12 months demonstrated a mean ± SD percentage change of 0.25% ± 1.28% in the alendronate group and −1.39% ± 1.33% in the placebo group. The study^(^ ^34^ ^)^ that reported on change in BMD at the distal radius at 12 months demonstrated a mean ± SD percentage change of 0.40% ± 3.39% in the alendronate group and −1.24% ± 4.25% in the placebo group.

**Fig. 3:**
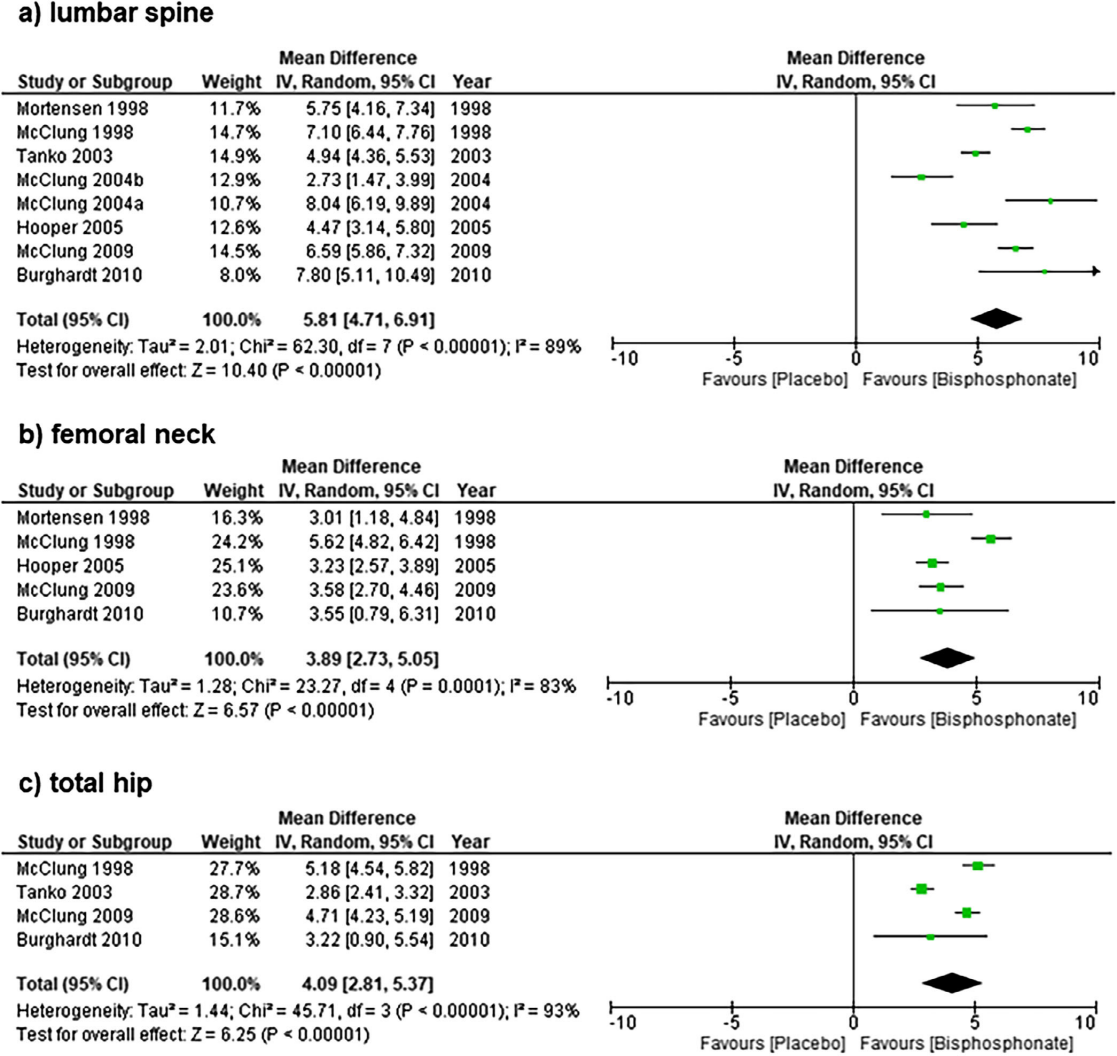
Mean (95% CI) percent change in lumbar spine BMD (Fig. 3a) was 5.81% (95% CI, 4.71%–6.91%) among eight studies over follow‐up intervals of 24,() 36,() and 72() months. Percent change in femoral neck BMD (Fig. 3b) was 3.89% (95% CI, 2.73%–5.05%) among five studies with follow‐up ranging from 24() to 36() months, and percent change in total hip BMD (Fig. 3c) was 4.09% (95% CI, 2.81%–5.37%) for four studies ranging from 24() to 36() months of follow‐up.

Studies assessing BMD changes over follow‐up intervals longer than 12 months are shown in Fig. 3. Mean (95% CI) percent change in lumbar spine BMD was 5.81% (95% CI, 4.71%–6.91%) among eight studies over follow‐up intervals of 24,^(^ ^27^, ^29^, ^31^, ^32^, ^33^, ^34^ ^)^ 36,^(^ ^26^ ^)^ and 72^(^ ^30^ ^)^ months. Percent change in femoral neck BMD was 3.89% (95% CI, 2.73%–5.05%) among five studies with follow‐up ranging from 24^(^ ^27^, ^32^, ^33^, ^34^ ^)^ to 36^(^ ^26^ ^)^ months, and percent change in total hip BMD was 4.09% (95% CI, 2.81%–5.37%) for four studies ranging from 24^(^ ^29^, ^32^, ^34^ ^)^ to 36^(^ ^26^ ^)^ months of follow‐up. Significant heterogeneity was observed for all BMD outcomes at follow‐up intervals longer than 12 months (*I* ^2^ 83%–93%). One study evaluated change in total body BMD after 36 months of follow‐up,^(^ ^26^ ^)^ reporting mean ± SD percent change of 0.66% ± 1.61% in the pooled alendronate group and −2.26% ± 1.65% in the placebo group. Two studies assessed change in BMD at the distal radius over a >12‐month follow‐up interval, with one reporting a percentage change of 0.20% ± 2.77% among women receiving alendronate and −2.21% ± 5.35% among women receiving placebo at 24 months,^(^ ^34^ ^)^ the other demonstrating changes of −1.60% ± 2.30% and −3.85% ± 3.06% among alendronate and placebo recipients, respectively, at 36 months.^(^ ^26^ ^)^

Results of subgroup analyses (data not shown) were comparable for the different types of bisphosphonates and did not explain the observed heterogeneity between studies over 12 months of follow‐up. Removal of 12‐month lumbar spine results from the trial that had not, to our knowledge, undergone peer review^(^ ^40^ ^)^ did not alter the meta‐analysis results at this site. For studies of >12‐months duration, subgroup analyses demonstrated lower heterogeneity for studies of alendronate (*I* ^2^ 0%–61%) and risedronate (*I* ^2^ 0%–32%). As no studies included women with established osteoporosis, we did not perform subgroup analyses for baseline BMD (ie, osteoporosis or not). We performed an exploratory subgroup analysis to determine whether menopausal status (perimenopause versus <5 years postmenopausal) contributed to heterogeneity. BMD changes and heterogeneity were similar for studies of perimenopausal and early postmenopausal women.

### Bone turnover marker outcomes

**Fig. 4:**
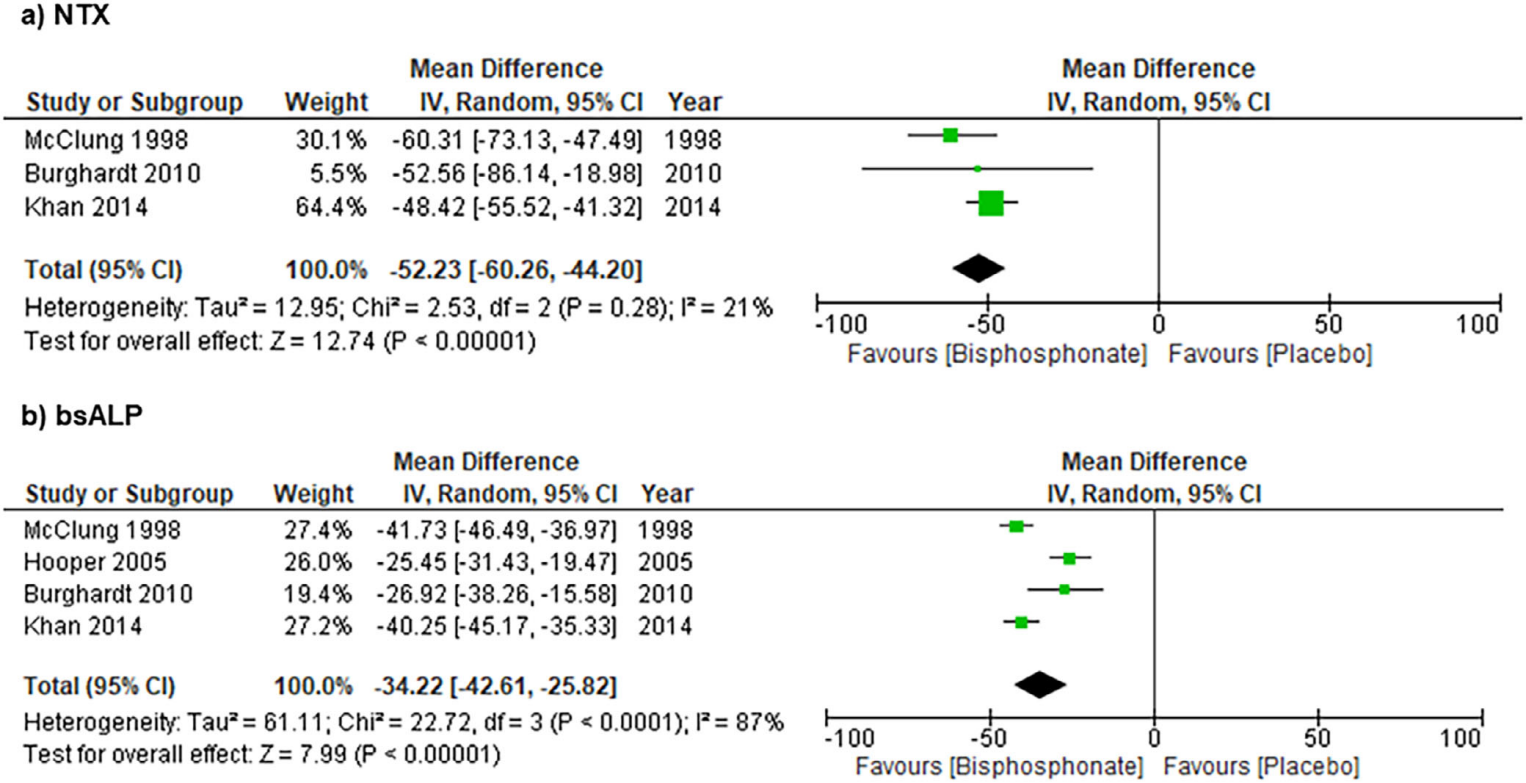
Effects of 12 months of treatment with nitrogen‐containing bisphosphonates versus placebo in early menopausal women on percent change in (*A*) urinary N‐telopeptide (NTX), and (*B*) bone‐specific alkaline phosphatase (bsALP); assessed using an inverse variance (IV) random‐effects meta‐analysis.

Changes in bone turnover markers are presented in Table 3, and pooled analyses for NTX and bsALP at 12 months are shown in Fig. 4. Pooled analyses demonstrated a 12‐month mean (95% CI) percent change (bisphosphonate – placebo) of −52.23% (95% CI, −60.26% to −44.20%) for NTX (Fig. 4a) and −34.22% (95% CI, −42.61% to −25.82%) for bsALP (Fig. 4b). Heterogeneity was low for NTX (*I* ^2^ = 21%) and high for bsALP (*I* ^2^ = 87%).

**Table 3:** Summary of Results from Trials Assessing the Effects of Nitrogen‐Containing Bisphosphonates on Bone Turnover Markers in Early Menopausal Women

Trial	Comparison	Time	CTX % change (SD)	NTX % change (SD)	bsALP % change (SD)
McClung and colleagues^(^ ^26^ ^)^ (1998) Lehmann and colleagues^(^ ^38^ ^)^ (2002) (substudy)	ALN 5–10 mg/day^a^ PO versus PBO	M12	‐	ALN: −68.9 (64.3)^b^ PBO: −8.6 (41.7)^b^	ALN: −31.7 (14.2)^b^ PBO: 10.1 (20.7)^b^
		M24	ALN: −65.1 (13.3) PBO: −16.5 (15.5)	–	–
Tanko and colleagues^(^ ^29^ ^)^ (2003)	IBN 20 mg/week PO versus PBO	M24	IBN: −41.7 (13.3)^b^ PBO: +7.4 (15.5)^b^	–	IBN: −9.4 (14.4)^b^ PBO: +2.9 (25.5)^b^
Hooper and colleagues^(^ ^33^ ^)^ (2005)	RIS 5 mg/day PO versus PBO	M12	–	–	RIS: −34.5 (14.4) PBO: 7.3 (25.5)
		M24	–	–	RIS: −20.9 (26.8) PBO: 3.0 (27.1)
Burghardt and colleagues^(^ ^34^ ^)^ (2010)	ALN 70 mg/week PO versus PBO	M12	–	ALN: −62.0 (64.3) PBO: −9.4 (41.7)	ALN: −36.4 (14.2) PBO: −9.5 (20.7)
		M24	–	ALN: −57.4 (34.7) PBO: −19.3 (29.4)	ALN: −37.0 (15.3) PBO: −11.8 (11.5)
Khan and colleagues^(^ ^24^ ^)^ (2014)	ALN 70 mg/week PO versus PBO	M12	–	ALN: −27.2 (11.2) PBO: 21.2 (11.2)	ALN: −37.8 (7.3) PBO: 2.5 (7.9)

^a^ Data pooled for ALN 5 mg and 10 mg groups.

^b^ SD imputed from similar study.

Abbreviation: ALN = alendronate; bsALP = bone specific alkaline phosphatase; CTX = C‐terminal telopeptide; IBN = ibandronate; M = month; NTX = N‐terminal telopeptide; PBO = placebo; PO = oral; RIS = risedronate; SD = standard deviation.

### Adverse events

Eight trials reported similar overall incidence of adverse events between bisphosphonate and placebo groups,^(^ ^24^, ^25^, ^26^, ^27^, ^28^, ^29^, ^30^, ^31^, ^33^, ^40^ ^)^ although McClung and colleagues^(^ ^26^ ^)^ observed a dose‐related increase in flatulence and odynophagia with alendronate therapy,^(^ ^26^ ^)^ Stakkestad and colleagues^(^ ^28^ ^)^ found that myalgias were more common in women receiving ibandronate 2 mg/day (22%) than placebo (4%), and Tanko and colleagues^(^ ^29^ ^)^ observed that the following adverse events were more frequent in the ibandronate arms than the placebo arm: back pain, bronchitis, sinusitis, gastroenteritis, bone fracture (number of fractures not reported), cystitis, gastrointestinal pain, myalgia, cholelithiasis, periodontal abscess. Hooper and colleagues^(^ ^33^ ^)^ reported a greater frequency of serious adverse events in women receiving placebo than those receiving risedronate. The 2009 study by McClung and colleagues^(^ ^32^ ^)^ reported similar rates of adverse events and serious adverse events across treatment groups, but did not present results specific to the early menopausal subgroup.^(39)^ Burghart and colleagues^(^ ^34^ ^)^ did not report adverse events.

### Reporting bias

Because all pooled analyses contained fewer than 10 studies, formal assessment of reporting bias was not undertaken, consistent with recommendations from the Cochrane Handbook.^(^ ^36^ ^)^

### Certainty assessment

In accordance with the GRADE framework for assessing strength of evidence, we concluded with moderate certainty that, compared to placebo, nitrogen‐containing bisphosphonates improve BMD at the lumbar spine, femoral neck, and total hip in perimenopausal and early menopausal women. Certainty was downgraded from high to moderate on the basis of between‐study heterogeneity and potential publication bias, although we judged that the magnitude of effect was large enough that there was likely to be a clinically significant effect despite the potential for residual confounding. Although only a small number of studies reported on changes in bone turnover markers, because a large lowering effect was consistent across studies, we concluded with moderate certainty that nitrogen‐containing bisphosphonates reduce bone turnover markers in perimenopausal and early menopausal women. We judged that existing controlled trials provide insufficient evidence to determine whether nitrogen‐containing bisphosphonates have a beneficial effect on fracture incidence in this population.

## Discussion

The vast majority of clinical trials assessing the efficacy of pharmacologic osteoporosis treatments to prevent fracture have been conducted in older women (average age >60 years) who have either established osteoporosis (ie, *T*‐score ≤ −2.5 at the spine or hip), high 10‐year absolute fracture risk (ie, ≥20%), or a prior fragility fracture.^(^ ^41^, ^42^ ^)^ These studies have demonstrated that nitrogen‐containing bisphosphonate therapy can reduce the risk of vertebral fracture by 50% to 70%, nonvertebral fracture by 20% to 30%, and hip fracture by 20% to 40%^(^ ^41^ ^)^ in older, high‐risk individuals. However, in addition to being approved for the use of osteoporosis *treatment*, bisphosphonates are also approved for osteoporosis *prevention*.^(^ ^10^ ^)^ Because the majority of bone loss occurs in the perimenopausal and early postmenopausal period,^(^ ^3^, ^4^, ^5^ ^)^ judicious use of bisphosphonates during this timeframe may prevent bone loss and reduce the future burden of osteoporosis.^(^ ^21^ ^)^ Because early menopausal women are at lower short‐term risk of fracture, there are fewer studies of bisphosphonates in this population, and existing studies were not designed to evaluate fracture incidence. We identified only three RCTs conducted in early menopausal women that reported fracture incidence.^(^ ^24^, ^27^, ^33^ ^)^ None of these trials considered fractures as a prespecified outcome, and fractures occurred infrequently in each study. Therefore, it is not possible to conclude whether bisphosphonates reduce fracture risk in the early menopausal population.

We found that bisphosphonates were effective in improving BMD at the spine and hip when administered in early menopause. Importantly, in a meta‐regression analysis of older adults treated with osteoporosis medication, improvements in BMD correlated with reduced fracture risk.^(^ ^41^ ^)^ Specifically, a 4% improvement in total hip BMD—similar to the change in BMD we observed in the present meta‐analysis over follow‐up intervals of 24 to 72 months—corresponded to a 51% reduction in vertebral fracture risk, a 16% reduction in nonvertebral fracture, and a 29% reduction in hip fracture. A 6% improvement in lumbar spine BMD—also comparable to what we observed in the present meta‐analysis—was associated with reductions of approximately 50% for vertebral fracture, 20% for nonvertebral fracture, and 30% for hip fracture. In general, the BMD changes observed in the present meta‐analysis were comparable to BMD changes in clinical trials of older women, which have demonstrated improvements of 3.5% to 7.6% at the lumbar spine, 2.1% to 5.1% at the femoral neck, and 2.1% to 6.4% at the total hip.^(^ ^41^ ^)^ This suggests that bisphosphonate therapy in early menopause may have a future fracture lowering effect.

Data from the Study of Women's Health Across the Nation (SWAN)^(^ ^43^ ^)^ also support the hypothesis that preservation of BMD around the time of menopause could lower long‐term fracture risk. In the SWAN cohort, rapid decline in lumbar spine BMD around the menopausal transition predicted future facture irrespective of baseline BMD.^(^ ^43^ ^)^ Furthermore, urinary NTX levels in SWAN participants were observed to rise sharply approximately 2 years prior to the final menstrual period, peaking 1 to 1.5 years after the final period and declining modestly from 2 to 6 years after menopause, indicating that bone resorption is maximized during the 2 years before and after the final menstrual period.^(^ ^22^ ^)^ In SWAN, a greater rate of increase of urinary NTX during the menopausal transition was associated with an increased risk of fracture.^(^ ^44^ ^)^ Bisphosphonates are deposited preferentially at sites of increased bone turnover^(^ ^23^ ^)^ and their long half‐life in bone^(^ ^17^ ^)^ means that either a short course or intermittent dosing might prevent the microstructural alterations—such as reduced trabecular number and increased trabecular spacing^(^ ^45^ ^)^—that accompany the high turnover state in perimenopause and the early postmenopausal period. In the present review, we observed sustained reductions in markers of bone resorption with bisphosphonate therapy, indicating that nitrogen‐containing bisphosphonates can suppress the usual increase in bone turnover in early menopause. Corroborating existing empirical evidence, a simulation study has indicated that an osteoporosis prevention strategy involving very infrequent infusions of zoledronate at 5‐year intervals, beginning at around age 50 years, could substantially reduce long‐term fracture risk and lower the population burden of osteoporosis.

The results of this systematic review and meta‐analysis should be considered in the context of several limitations. First, we limited our literature search to randomized controlled trials. The small number of trials meeting inclusion criteria—most of which did not assess fracture incidence and did not extend beyond 24 months—precludes our ability to determine whether bisphosphonate therapy reduces long‐term fracture risk when administered in early menopause. Second, although the included studies consistently demonstrated that bisphosphonate therapy improves BMD in early menopausal women irrespective of bisphosphonate type and menopausal status (perimenopause or early postmenopause), there was a wide variation in the degree of BMD improvement among included studies that could not be explained via subgroup analyses. Although reasons for the observed between‐study heterogeneity require further investigations, the consistent beneficial effect of nitrogen‐containing bisphosphonates across all included studies allows us to conclude with moderate certainty that these agents improve BMD in early menopause. Third, although there were not enough studies included in this review to justify formal testing for publication bias, the fact that more than half of studies had missing outcome data and all studies were industry‐funded suggests a bias toward the selective reporting of positive results. Importantly, the literature search was limited to English‐language studies, and the vast majority of participants in the included trials which reported on ethnicity were white, meaning that the results of this meta‐analysis may have limited generalizability within multiethnic populations.

### Conclusions

This systematic review and meta‐analysis demonstrates that nitrogen‐containing bisphosphonates improve BMD and reduce bone turnover markers compared to placebo in early menopausal women. The effects of bisphosphonates in this population are comparable those in older women at higher near‐term fracture risk, in whom similar BMD improvements have been associated with reduced fracture incidence.^(^ ^41^ ^)^ The implication of our findings is that an osteoporosis prevention strategy involving judicious use of bisphosphonate medications around the time of menopause can be reasonably expected to preserve bone mass, delay the development of osteoporosis, and lower long‐term fracture risk. Such a strategy could substantially reduce the burden of osteoporosis in the aging population. Well‐designed trials assessing the efficacy of bisphosphonate therapy as a potential osteoporosis and fracture prevention strategy in a multiethnic cohort of early menopausal women are therefore warranted.

## Author Contributions

**Aria Ahadzadeh Ardebili:** Conceptualization; data curation; investigation; methodology; project administration; writing – original draft. **Timothy Fu:** Data curation; investigation; project administration. **Nicole Dunnewold:** Conceptualization; data curation; investigation; methodology; project administration. **Fariba Aghajafari:** Conceptualization; data curation; formal analysis; investigation; methodology; supervision; writing – original draft; writing – review and editing. **Emma Billington:** Conceptualization; data curation; formal analysis; funding acquisition; investigation; methodology; project administration; supervision; writing – original draft; writing – review and editing.

## Disclosures

AAA, TF, ND, FA and EOB report no conflicts of interest.

### Peer Review

The peer review history for this article is available at https://www.webofscience.com/api/gateway/wos/peer‐review/10.1002/jbm4.10748.

## Supporting information


**Data S1.** Supporting Information

## Data Availability

Data extraction forms have not been published but are available from the corresponding author upon reasonable request.
