# Supplementary material for: Bisphosphonates Preserve Bone Mineral Density and Suppress Bone Turnover Markers in Early Menopausal Women: A Systematic Review and Meta‐Analysis of Randomized Trials
Source: JBMR Plus. 2023 Apr 14;7(6):e10748. doi: 10.1002/jbm4.10748 (PMC10241086; doi:10.1002/jbm4.10748)
Supplement: Supplementary file 1 — Data S1. Supporting Information [file JBM4-7-e10748-s001.pdf]

Bisphosphonates preserve bone mineral density and suppress bone turnover markers in early menopausal women: A systematic review and meta-analysis of randomized trials

Supplementary Materials: Search Strategy

Ovid®
Wolters Kluwer
My Account Ask a Librarian Support & Training Help Feedback Logged in as Nicole Dunnewold Logoff
Search Journals Books Multimedia My Workspace EBP Tools

Embase <1974 to 2020 April 14>

#	Searches	Results	Type
1	bisphosphonic acid derivative/ or alendronic acid/ or ibandronic acid/ or neridronic acid/ or pamidronic acid/ or risedronic acid/ or zoledronic acid/	57428	Advanced
2	(bisphosphon* or diphosphon*).tw,kw.	31508	Advanced
3	(alendronic acid or alendronate* or Fosamax).tw,kw.	8520	Advanced
4	(ibandronic acid or ibandronate* or Boniva or Bonviva).tw,kw.	2285	Advanced
5	(neridronic acid or neridronate* or Nerixia).tw,kw.	183	Advanced
6	(pamidronic acid or pamidronate* or amidronate or Aredia).tw,kw.	4283	Advanced
7	(risedronic acid or risdronate* or acrel or actonel).tw,kw.	931	Advanced
8	(zoledronic acid or zoledronate or zometa or aclasta or reclast).tw,kw.	9182	Advanced
9	or/1-8	64790	Advanced
10	osteoporosis/ or involutional osteoporosis/ or postmenopause osteoporosis/ or primary osteoporosis/	123355	Advanced
11	bone demineralization/	2746	Advanced
12	bone density/ or bone densitometer/ or bone densitometry/ or ultrasound bone densitometer/ or x ray bone densitometer/ or dual energy x ray absorptiometry/ or photon absorptiometry/	116794	Advanced
13	osteolysis/	69061	Advanced
14	bone remodeling/	30768	Advanced
15	exp fracture/	281214	Advanced
16	bone mass/	27320	Advanced
17	bone turnover/	18072	Advanced
18	fracture*.tw,kw.	297743	Advanced
19	(bone* adj2 (densi* or content* or resorption or loss or mass or remodel* or turnover or turn-over or break or broken or decalcif* or deminerali*)).tw,kw.	163243	Advanced
20	BMD test*.tw,kw.	819	Advanced
21	(osteoporos?s or osteolys?s).tw,kw.	119477	Advanced
22	((dpx or dual energy or dual-energy or dual-photon or photon or single-photon or xray or x-ray or x ray or dexa or dxa or ultrasound or ultra-sound) adj2 (absorptiomet* or scan* or densitomet* or photodensitomet*)).tw,kw.	62127	Advanced
23	or/10-22	630457	Advanced
24	"menopause and climacterium"/ or climacterium/ or early menopause/ or menopause/ or postmenopause/	107877	Advanced
25	postmenopause osteoporosis/	14053	Advanced
26	(menopau* or perimenopaus* or peri-menopaus* or postmenopaus* or post-menopaus* or climacter*).tw,kw.	133701	Advanced
27	24 or 25 or 26	159761	Advanced
28	9 and 23 and 27	9750	Advanced
29	exp clinical trial/	1482270	Advanced
30	randomi?ed.ti,ab.	881168	Advanced
31	placebo.ti,ab.	303955	Advanced
32	dt.fs.	3700866	Advanced
33	randomly.ti,ab.	438606	Advanced
34	trial.ti,ab.	843475	Advanced
35	groups.ti,ab.	2846657	Advanced
36	29 or 30 or 31 or 32 or 33 or 34 or 35	7397559	Advanced
37	exp animal/	25372193	Advanced
38	human/	20614807	Advanced
39	37 not (37 and 38)	4757386	Advanced
40	36 not 39	6581577	Advanced
41	28 and 40	8119	Advanced
42	28 and 40	8119	Advanced
43	limit 42 to conference abstracts	1043	Advanced
44	42 not 43	7076	Advanced

English Français Italiano Deutsch 日本語 繁體中文 Español 简体中文 한국어
About Us Contact Us Privacy Policy Terms of Use
© 2020 Ovid Technologies, Inc. All rights reserved. OvidUI_04.05.00.021, SourceID d9d3a66c581497ad545b4e3f15aea4a623901eb3

Ovid®

Wolters Kluwer

My Account | Ask a Librarian | Support & Training | Help | Feedback | Logged in as Nicole Dunnewold | Logoff

Search | Journals | Books | Multimedia | My Workspace | EBP Tools

Ovid MEDLINE(R) and Epub Ahead of Print, In-Process & Other Non-Indexed Citations and Daily <1946 to April 14, 2020>

#	Searches	Results	Type
1	diphosphonates/ or alendronate/ or ibandronic acid/ or pamidronate/ or risedronic acid/ or zoledronic acid/	19535	Advanced
2	(Bisphosphon* or diphosphon*).tw,kf.	20991	Advanced
3	(alendronic acid or alendronate* or Fosamax).tw,kf.	4708	Advanced
4	(ibandronic acid or ibandronate* or Boniva or Bonviva).tw,kf.	1038	Advanced
5	(neridronic acid or neridronate* or Nerixia).tw,kf.	118	Advanced
6	(pamidronic acid or pamidronate* or amidronate or Aredia).tw,kf.	2493	Advanced
7	(risedronic acid or risdronate* or acrel or actonel).tw,kf.	90	Advanced
8	(zoledronic acid or zoledronate or zometa or aclasta or reclast).tw,kf.	4712	Advanced
9	or/1-8	30790	Advanced
10	Osteoporosis, Postmenopausal/ or Osteoporosis/	55144	Advanced
11	Bone Density/	52650	Advanced
12	bone resorption/ or osteolysis/	27615	Advanced
13	bone remodeling/	12554	Advanced
14	bone demineralization, pathologic/ or decalcification, pathologic/	651	Advanced
15	exp Fractures, Bone/	182177	Advanced
16	Absorptiometry, Photon/	22478	Advanced
17	fracture*.tw,kf.	256851	Advanced
18	(bone* adj2 (densi* or content* or resorption or loss or mass or remodel* or turnover or turn-over or break or broken or decalcif* or deminerali*)).tw,kf.	120572	Advanced
19	BMD test*.tw,kf.	426	Advanced
20	(osteoporos?s or osteolys?s).tw,kf.	76822	Advanced
21	((dpx or dual energy or dual-energy or dual-photon or photon or single-photon or xray or x-ray or x ray or dexa or dxa or ultrasound or ultra-sound) adj2 (absorptiomet* or scan* or densitomet* or photodensitomet*)).tw,kf.	43456	Advanced
22	or/10-21	468470	Advanced
23	menopause/ or perimenopause/ or postmenopause/ or osteoporosis, postmenopausal/	61971	Advanced
24	(menopaus* or perimenopaus* or peri-menopaus* or postmenopaus* or post-menopaus* or climacter*).tw,kf.	93594	Advanced
25	or/23-24	107507	Advanced
26	9 and 22 and 25	4355	Advanced
27	randomized controlled trial.pt.	503984	Advanced
28	controlled clinical trial.pt.	93621	Advanced
29	randomi?ed.ti,ab.	614077	Advanced
30	placebo.ti,ab.	212526	Advanced
31	dt.fs.	2195231	Advanced
32	randomly.ti,ab.	332054	Advanced
33	trial.ti,ab.	586233	Advanced
34	groups.ti,ab.	2059820	Advanced
35	or/27-34	4754901	Advanced
36	exp animals/	23100450	Advanced
37	humans/	18409620	Advanced
38	36 not (36 and 37)	4690830	Advanced
39	35 not 38	4127828	Advanced
40	26 and 39	3407	Advanced

English | Français | Italiano | Deutsch | 日本語 | 繁體中文 | Español | 简体中文 | 한국어

About Us | Contact Us | Privacy Policy | Terms of Use

© 2020 Ovid Technologies, Inc. All rights reserved. OvidUI_04.05.00.021, SourceID d9d3a66c581497ad545b4e3f15aea4a623901eb3

Ovid®
Wolters Kluwer
My Account | Ask a Librarian | Support & Training | Help | Feedback | Logged in as Nicole Dunnewold | Logoff

Search | Journals | Books | Multimedia | My Workspace | EBP Tools

EBM Reviews - Cochrane Central Register of Controlled Trials

#	Searches	Results	Type
1	diphosphonates/ or alendronate/	1841	Advanced
2	(bisphosphon* or diphosphon*).tw,kw.	2554	Advanced
3	(alendronic acid or alendronate* or Fosamax).tw,kw.	1487	Advanced
4	(ibandronic acid or ibandronate* or Boniva or Bonviva).tw,kw.	465	Advanced
5	(neridronic acid or neridronate* or Nerixia).tw,kw.	51	Advanced
6	(pamidronic acid or pamidronate* or amidronate).tw,kw.	540	Advanced
7	(risedronic acid or risdronate* or acrel or actonel).tw,kw.	115	Advanced
8	(zoledronic acid or zoledronate or zometa or aclasta or reclast).tw,kw.	1598	Advanced
9	or/1-8	5278	Advanced
10	osteoporosis/ or osteoporosis, postmenopausal/	4027	Advanced
11	bone demineralization, pathologic/ or decalcification, pathologic/	19	Advanced
12	bone resorption/ or osteolysis/	979	Advanced
13	bone remodeling/	867	Advanced
14	bone density/	4535	Advanced
15	exp Fractures, Bone/	5686	Advanced
16	absorptiometry, photon/	1882	Advanced
17	fracture*.tw,kw.	21400	Advanced
18	(bone* adj2 (densi* or content* or resorption or loss or mass or remodel* or turnover or turn-over or break or broken or decalcif* or deminerali*)).tw,kw.	16891	Advanced
19	BMD test*.tw,kw.	91	Advanced
20	(osteoporos?s or osteolys?s).tw,kw.	10321	Advanced
21	((dpx or dual energy or dual-energy or dual-photon or photon or single-photon or xray or x-ray or x ray or dexa or dxa or ultrasound or ultra-sound) adj2 (absorptiomet* or scan* or densitomet* or photodensitomet*)).tw,kw.	7701	Advanced
22	10 or 11 or 12 or 13 or 14 or 15 or 16 or 17 or 18 or 19 or 20 or 21	41572	Advanced
23	climacteric/ or menopause/ or menopause, premature/ or perimenopause/ or postmenopause/	6378	Advanced
24	Osteoporosis, Postmenopausal/	2002	Advanced
25	(menopau* or perimenopaus* or peri-menopaus* or postmenopaus* or post-menopaus* or climacter*).tw,kw.	26288	Advanced
26	23 or 24 or 25	26974	Advanced
27	9 and 22 and 26	1689	Advanced

English Français Italiano Deutsch 日本語 繁體中文 Español 简体中文 한국어

About Us | Contact Us | Privacy Policy | Terms of Use

© 2020 Ovid Technologies, Inc. All rights reserved. OvidUI_04.05.00.021, SourceID d9d3a66c581497ad545b4e3f15aea4a623901eb3

MY EBSCOhost

Wednesday, April 15, 2020 4:51:11 PM

#	Query	Limiters/Expanders	Last Run Via	Results
S28	(((MH "Random Assignment") or (MH "Random Sample+") or (MH "Crossover Design") or (MH "Clinical Trials+") or (MH "Comparative Studies") or (MH "Control (Research)+") or (MH "Control Group") or (MH "Factorial Design") or (MH "Quasi-Experimental Studies+") or (MH "Placebos") or (MH "Meta Analysis") or (MH "Sample Size") or (MH "Research, Nursing") or (MH "Research Question") or (MH "Research Methodology+") or (MH "Evaluation Research+") or (MH "Concurrent Prospective Studies") or (MH "Prospective Studies") or (MH "Nursing Practice, Research-Based") or (MH "Solomon Four-Group Design") or (MH "One-Shot Case Study") or (MH "Pretest-Posttest Design+") or (MH "Static Group Comparison") or (MH "Study Design") or (MH "Clinical Research+")) or (clinical nursing research or random* or cross?over or placebo* or control* or factorial or sham* or	Search modes - Find all my search terms	Interface - EBSCOhost Research Databases<br>Search Screen - Advanced Search<br>Database - CINAHL Plus with Full Text	1,342

	meta?analy* or systematic review* or blind* or mask* or trial*)) AND (S26 AND S27)			
S27	((MH "Random Assignment") or (MH "Random Sample+") or (MH "Crossover Design") or (MH "Clinical Trials+") or (MH "Comparative Studies") or (MH "Control (Research)+") or (MH "Control Group") or (MH "Factorial Design") or (MH "Quasi-Experimental Studies+") or (MH "Placebos") or (MH "Meta Analysis") or (MH "Sample Size") or (MH "Research, Nursing") or (MH "Research Question") or (MH "Research Methodology+") or (MH "Evaluation Research+") or (MH "Concurrent Prospective Studies") or (MH "Prospective Studies") or (MH "Nursing Practice, Research-Based") or (MH "Solomon Four-Group Design") or (MH "One-Shot Case Study") or (MH "Pretest-Posttest Design+") or (MH "Static Group Comparison") or (MH "Study Design") or (MH "Clinical Research+")) or (clinical nursing research or random* or cross?over or placebo* or control* or factorial or sham* or meta?analy* or systematic review* or blind* or mask* or trial*)	Search modes - Find all my search terms	Interface - EBSCOhost Research Databases<br>Search Screen - Advanced Search<br>Database - CINAHL Plus with Full Text	3,153,045

S26	(S22 OR S23 OR S24) AND (S9 AND S21 AND S25)	Search modes - Find all my search terms	Interface - EBSCOhost Research Databases<br>Search Screen - Advanced Search<br>Database - CINAHL Plus with Full Text	1,636
S25	S22 OR S23 OR S24	Search modes - Find all my search terms	Interface - EBSCOhost Research Databases<br>Search Screen - Advanced Search<br>Database - CINAHL Plus with Full Text	35,888
S24	TI ((menopau* or perimenopaus* or perimenopaus* or postmenopaus* or postmenopaus* or climacter*)) OR AB ((menopau* or perimenopaus* or perimenopaus* or postmenopaus* or postmenopaus* or climacter*))	Search modes - Find all my search terms	Interface - EBSCOhost Research Databases<br>Search Screen - Advanced Search<br>Database - CINAHL Plus with Full Text	29,418
S23	(MH "Perimenopausal Symptoms") OR (MH "Postmenopausal Disorders")	Search modes - Find all my search terms	Interface - EBSCOhost Research Databases<br>Search Screen - Advanced Search<br>Database - CINAHL Plus with Full Text	3,147
S22	(MH "Postmenopause") OR (MH "Perimenopause") OR (MH "Menopause, Premature") OR (MH "Menopause") OR (MH "Climacteric")	Search modes - Find all my search terms	Interface - EBSCOhost Research Databases<br>Search Screen - Advanced Search<br>Database - CINAHL Plus with Full Text	22,235
S21	S10 OR S11 OR S12 OR S13 OR S14 OR S15 OR S16 OR S17 OR S18 OR S19 OR S20	Search modes - Find all my search terms	Interface - EBSCOhost Research Databases<br>Search Screen - Advanced Search<br>Database - CINAHL Plus with Full Text	136,758

S20	TI (((dpx or dual energy or dual-energy or dualphoton or photon or single-photon or xray or xray or x ray or dexa or dxa or ultrasound or ultrasound) N2 (absorptiomet* or scan* or densitomet* or photodensitomet*))) OR AB (((dpx or dual energy or dual-energy or dualphoton or photon or single-photon or xray or xray or x ray or dexa or dxa or ultrasound or ultrasound) N2 (absorptiomet* or scan* or densitomet* or photodensitomet*)))	Search modes - Find all my search terms	Interface - EBSCOhost Research Databases<br>Search Screen - Advanced Search<br>Database - CINAHL Plus with Full Text	11,847
S19	TI ((osteoporos?s or osteolys?s)) OR AB ((osteoporos?s or osteolys?s))	Search modes - Find all my search terms	Interface - EBSCOhost Research Databases<br>Search Screen - Advanced Search<br>Database - CINAHL Plus with Full Text	21,296
S18	TI BMD test* OR AB BMD test*	Search modes - Find all my search terms	Interface - EBSCOhost Research Databases<br>Search Screen - Advanced Search<br>Database - CINAHL Plus with Full Text	1,373
S17	TI ((bone* N2 (densi* or content* or resorption or loss or mass or remodel* or turnover or turn-over or break or broken or decalcif* or demineral i*))) OR AB ((bone* N2 (densi* or content* or resorption or loss or mass or remodel* or turnover or turn-over or break or	Search modes - Find all my search terms	Interface - EBSCOhost Research Databases<br>Search Screen - Advanced Search<br>Database - CINAHL Plus with Full Text	28,759

	broken or decalcif* or deminerali*)))			
S16	TI fracture* OR AB fracture*	Search modes - Find all my search terms	Interface - EBSCOhost Research Databases<br>Search Screen - Advanced Search<br>Database - CINAHL Plus with Full Text	73,017
S15	(MH "Absorptiometry, Photon")	Search modes - Find all my search terms	Interface - EBSCOhost Research Databases<br>Search Screen - Advanced Search<br>Database - CINAHL Plus with Full Text	10,599
S14	(MH "Fractures+")	Search modes - Find all my search terms	Interface - EBSCOhost Research Databases<br>Search Screen - Advanced Search<br>Database - CINAHL Plus with Full Text	60,559
S13	(MH "Bone Density")	Search modes - Find all my search terms	Interface - EBSCOhost Research Databases<br>Search Screen - Advanced Search<br>Database - CINAHL Plus with Full Text	18,817
S12	(MH "Bone Remodeling")	Search modes - Find all my search terms	Interface - EBSCOhost Research Databases<br>Search Screen - Advanced Search<br>Database - CINAHL Plus with Full Text	2,856
S11	(MH "Osteolysis") OR (MH "Bone Resorption")	Search modes - Find all my search terms	Interface - EBSCOhost Research Databases<br>Search Screen - Advanced Search<br>Database - CINAHL Plus with Full Text	7,099
S10	(MH "Osteoporosis") OR (MH "Osteoporotic Fractures")	Search modes - Find all my search terms	Interface - EBSCOhost Research Databases<br>Search Screen - Advanced Search	24,242

			Database - CINAHL Plus with Full Text	
S9	S1 OR S2 OR S3 OR S4 OR S5 OR S6 OR S7 OR S8	Search modes - Find all my search terms	Interface - EBSCOhost Research Databases<br>Search Screen - Advanced Search<br>Database - CINAHL Plus with Full Text	11,470
S8	TI ((zoledronic acid or zoledronate or zometa or aclasta or reclast)) OR AB ((zoledronic acid or zoledronate or zometa or aclasta or reclast))	Search modes - Find all my search terms	Interface - EBSCOhost Research Databases<br>Search Screen - Advanced Search<br>Database - CINAHL Plus with Full Text	1,569
S7	TI ((risedronic acid or risdronate* or acrel or actonel)) OR AB ((risedronic acid or risdronate* or acrel or actonel))	Search modes - Find all my search terms	Interface - EBSCOhost Research Databases<br>Search Screen - Advanced Search<br>Database - CINAHL Plus with Full Text	46
S6	TI ((pamidronic acid or pamidronate* or amidronate)) OR AB ((pamidronic acid or pamidronate* or amidronate))	Search modes - Find all my search terms	Interface - EBSCOhost Research Databases<br>Search Screen - Advanced Search<br>Database - CINAHL Plus with Full Text	508
S5	TI ((neridronic acid or neridronate* or Nerixia)) OR AB ((neridronic acid or neridronate* or Nerixia))	Search modes - Find all my search terms	Interface - EBSCOhost Research Databases<br>Search Screen - Advanced Search<br>Database - CINAHL Plus with Full Text	25
S4	TI ((ibandronic acid or ibandronate* or Boniva or Bonviva)) OR AB ((ibandronic acid or ibandronate* or Boniva or Bonviva))	Search modes - Find all my search terms	Interface - EBSCOhost Research Databases<br>Search Screen - Advanced Search<br>Database - CINAHL Plus with Full Text	352
S3	TI ((alendronic acid or alendronate* or Fosamax)) OR AB ((alendronic acid	Search modes - Find all my search terms	Interface - EBSCOhost Research Databases<br>Search Screen - Advanced Search	1,398

	or alendronate* or Fosamax))		Database - CINAHL Plus with Full Text	
S2	TI ((bisphosphon* or diphosphon*)) OR AB ((bisphosphon* or diphosphon*))	Search modes - Find all my search terms	Interface - EBSCOhost Research Databases<br>Search Screen - Advanced Search<br>Database - CINAHL Plus with Full Text	5,520
S1	(MH "Diphosphonates") OR (MH "Alendronate") OR (MH "Ibandronate Sodium") OR (MH "Risedronate") OR (MH "Zoledronic Acid")	Search modes - Find all my search terms	Interface - EBSCOhost Research Databases<br>Search Screen - Advanced Search<br>Database - CINAHL Plus with Full Text	9,477

Web of Science

Clarivate Analytics

Search History

Web of Science Core Collection

Save History / Create Alert | Open Saved History

Combine Sets: AND OR Combine | Delete Sets: Select All Delete

Set	Results	Query	Edit Sets
# 9	43	#6 AND #5 AND #1<br>**Refined by: PUBLICATION YEARS:** (2020 OR 2019 OR 2018 OR 2017) AND **DOCUMENT TYPES:** (PROCEEDINGS PAPER OR MEETING ABSTRACT)<br>*Indexes=SCI-EXPANDED, SSCI, A&HCI, CPCI-S, CPCI-SSH, ESCI Timespan=All years*	
# 8	1,044	#6 AND #5 AND #1<br>**Refined by: PUBLICATION YEARS:** (2020 OR 2019 OR 2018 OR 2017)<br>*Indexes=SCI-EXPANDED, SSCI, A&HCI, CPCI-S, CPCI-SSH, ESCI Timespan=All years*	
# 7	7,045	#6 AND #5 AND #1<br>*Indexes=SCI-EXPANDED, SSCI, A&HCI, CPCI-S, CPCI-SSH, ESCI Timespan=All years*	Edit
# 6	127,791	**TOPIC:** ((menopaus* or perimenopaus* or peri-menopaus* or postmenopaus* or post-menopaus* or climacter*))<br>*Indexes=SCI-EXPANDED, SSCI, A&HCI, CPCI-S, CPCI-SSH, ESCI Timespan=All years*	Edit
# 5	737,792	#4 OR #3 OR #2<br>*Indexes=SCI-EXPANDED, SSCI, A&HCI, CPCI-S, CPCI-SSH, ESCI Timespan=All years*	Edit
# 4	108,147	**TOPIC:** ((dpx NEAR/2 (absorptiomet* or scan* or densitomet* or photodensitomet*))) *OR* **TOPIC:** (("dual energy" NEAR/2 (absorptiomet* or scan* or densitomet* or photodensitomet*))) *OR* **TOPIC:** ((dual-energy NEAR/2 (absorptiomet* or scan* or densitomet* or photodensitomet*))) *OR* **TOPIC:** ((dual-photon NEAR/2 (absorptiomet* or scan* or densitomet* or photodensitomet*))) *OR* **TOPIC:** ((photon NEAR/2 (absorptiomet* or scan* or densitomet* or photodensitomet*))) *OR* **TOPIC:** ((single-photon NEAR/2 (absorptiomet* or scan* or densitomet* or photodensitomet*))) *OR* **TOPIC:** ((xray NEAR/2 (absorptiomet* or scan* or densitomet* or photodensitomet*))) *OR* **TOPIC:** ((x-ray NEAR/2 (absorptiomet* or scan* or densitomet* or photodensitomet*))) *OR* **TOPIC:** (("x ray" NEAR/2 (absorptiomet* or scan* or densitomet* or photodensitomet*))) *OR* **TOPIC:** ((dexa NEAR/2 (absorptiomet* or scan* or densitomet* or photodensitomet*))) *OR* **TOPIC:** ((dxa NEAR/2 (absorptiomet* or scan* or densitomet* or photodensitomet*))) *OR* **TOPIC:** ((ultrasound NEAR/2 (absorptiomet* or scan* or densitomet* or photodensitomet*))) *OR* **TOPIC:** ((ultra-sound NEAR/2 (absorptiomet* or scan* or densitomet* or photodensitomet*)))<br>*Indexes=SCI-EXPANDED, SSCI, A&HCI, CPCI-S, CPCI-SSH, ESCI Timespan=All years*	Edit
# 3	560,071	**TOPIC:** (fracture*) *OR* **TOPIC:** (BMD test*) *OR* **TOPIC:** ((osteoporos?s or osteolys?s))<br>*Indexes=SCI-EXPANDED, SSCI, A&HCI, CPCI-S, CPCI-SSH, ESCI Timespan=All years*	Edit
# 2	157,376	TS=(bone* NEAR/2 (densi* or content* or resorption or loss or mass or remodel* or turnover or turn-over or break or broken or decalcif* or demineral*))<br>*Indexes=SCI-EXPANDED, SSCI, A&HCI, CPCI-S, CPCI-SSH, ESCI Timespan=All years*	Edit
# 1	39,049	TS=(Bisphosphon* or diphosphon*) OR TS=(alendronic acid or alendronate* or Fosamax) OR TS=(ibandronic acid or ibandronate* or Boniva or Bonviva) OR TS=(neridronic acid or neridronate* or Nerixia) OR TS=(pamidronic acid or pamidronate* or amidronate) OR TS=(risedronic acid or risdronate* or acrel or actonel) OR TS=(zoledronic acid or zoledronate or zometa or aclasta or reclast)<br>*Indexes=SCI-EXPANDED, SSCI, A&HCI, CPCI-S, CPCI-SSH, ESCI Timespan=All years*	Edit

AND OR Combine | Select All Delete

Clarivate
Accelerating innovation

© 2020 Clarivate Copyright notice Terms of use Privacy statement Cookie policy

Sign up for the Web of Science newsletter Follow us
